# Poly[hemi(hexa­aqua­zinc) [[μ_2_-1,3-bis­(1,2,4-triazol-1-yl)methane](μ_2_-5-sulfonato­benzene-1,3-dicarboxyl­ato)zinc] sesquihydrate]

**DOI:** 10.1107/S1600536811022835

**Published:** 2011-06-18

**Authors:** Shang-Yuan Liu, Li Tian

**Affiliations:** a College of Chemistry, Tianjin Normal University, Tianjin 300387, People’s Republic of China

## Abstract

The title coordination polymer, {[Zn(H_2_O)_6_]_0.5_[Zn(C_8_H_3_O_7_S)(C_5_H_6_N_6_)]·1.5H_2_O}_*n*_, synthesized under hydro­thermal conditions, possesses a one-dimensional tube-like chain structure along [100], with octahedral [Zn(H_2_O)_6_]^2+^ groups (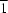 symmetry) trapped in the pores. The other Zn atom is five-coordinated in a highly distorted trigonal–biyramidal coordin­ation that is defined by two different N atoms from two 1,3-bis­(1,2,4-triazol-1-yl)methane (btrm) ligands and three carboxyl­ate O atoms from 5-sulfonato­benzene-1,3-dicarboxyl­ate ligands. The chains carry negative charges, whereas the free [Zn(H_2_O)_6_]^2+^ cations are positively charged. The [Zn(H_2_O)_6_]^2+^ cation is connected with the one-dimensional tubelike chain through weak classical O—H⋯O and O—H⋯N hydrogen-bonding inter­actions as well as through electrostatic inter­actions. One of the two uncoordinated water molecules exhibits half-occupancy.

## Related literature

For properties of organic–inorganic hybrid materials, see: Ishikava *et al.* (2003 ▶). One of the key steps in the preparation of polymeric transition metal complexes is to select multidentate bridging ligands or mixed multidentate ligands, see: Biradha *et al.* (2006 ▶).
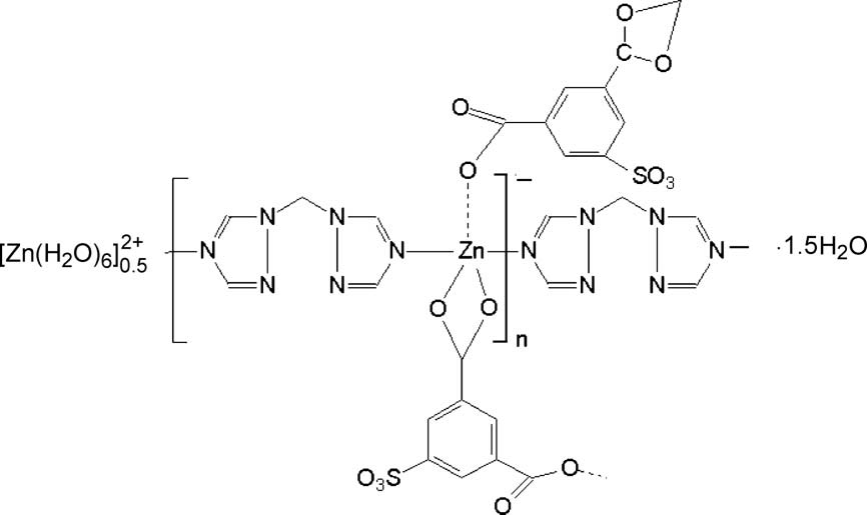


## Experimental

### 

#### Crystal data

[Zn(H_2_O)_6_]_0.5_[Zn(C_8_H_3_O_7_S)(C_5_H_6_N_6_)]·1.5H_2_O
*M* *_r_* = 571.48
Monoclinic, 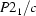

*a* = 10.2611 (3) Å
*b* = 16.9967 (4) Å
*c* = 11.4808 (3) Å
β = 93.812 (2)°
*V* = 1997.88 (9) Å^3^
*Z* = 4
Mo *K*α radiation
μ = 2.00 mm^−1^
*T* = 293 K
0.23 × 0.15 × 0.14 mm

#### Data collection

Bruker SuperNova Eos diffractometer
Absorption correction: multi-scan (*SADABS*; Bruker, 1997 ▶) *T*_min_ = 0.657, *T*_max_ = 0.767
7740 measured reflections
3524 independent reflections
2763 reflections with *I* > 2σ(*I*)
*R*_int_ = 0.030

#### Refinement

*R*[*F* ^2^ > 2σ(*F* ^2^)] = 0.049
*wR*(*F* ^2^) = 0.155
*S* = 1.11
3524 reflections
304 parameters
30 restraints
H-atom parameters constrained
Δρ_max_ = 1.67 e Å^−3^
Δρ_min_ = −0.90 e Å^−3^

### 

Data collection: *SMART* (Bruker, 1997 ▶); cell refinement: *SAINT* (Bruker, 1997 ▶); data reduction: *SAINT*; program(s) used to solve structure: *SHELXS97* (Sheldrick, 2008 ▶); program(s) used to refine structure: *SHELXL97* (Sheldrick, 2008 ▶); molecular graphics: *SHELXTL* (Sheldrick, 2008 ▶); software used to prepare material for publication: *SHELXTL*.

## Supplementary Material

Crystal structure: contains datablock(s) global, I. DOI: 10.1107/S1600536811022835/br2168sup1.cif

Structure factors: contains datablock(s) I. DOI: 10.1107/S1600536811022835/br2168Isup2.hkl

Additional supplementary materials:  crystallographic information; 3D view; checkCIF report

## Figures and Tables

**Table 1:** Hydrogen-bond geometry (Å, °)

*D*—H⋯*A*	*D*—H	H⋯*A*	*D*⋯*A*	*D*—H⋯*A*
O8—H8*A*⋯O3^i^	0.86	1.90	2.628 (7)	141
O8—H8*B*⋯O11	0.86	2.40	3.126 (7)	143
O8—H8*B*⋯O7^ii^	0.86	2.49	2.983 (7)	117
O9—H9*A*⋯O6^iii^	0.86	1.91	2.700 (7)	151
O9—H9*B*⋯O5^ii^	0.86	1.94	2.794 (7)	170
O10—H10*A*⋯O2	0.86	1.98	2.716 (7)	143
O10—H10*B*⋯O11	0.86	2.12	2.656 (7)	119
O10—H10*B*⋯O12	0.86	2.39	3.068 (7)	135
O11—H11*A*⋯O7^iii^	0.86	2.37	2.887 (7)	119
O11—H11*B*⋯N5	0.86	2.39	3.029 (7)	131

Symmetry codes: (i) 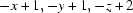; (ii) 
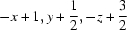
; (iii) 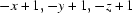.

**Fig. 1.**
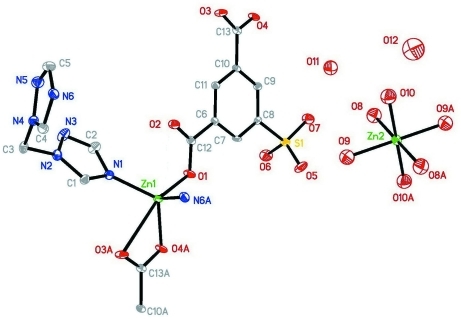
The coordination environments of Zn1 in the title compound.

**Fig. 2.**
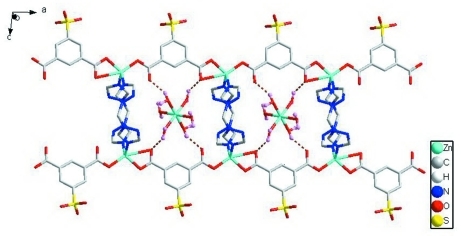
The one-dimensional chain of the title compound.

**Fig. 3.**
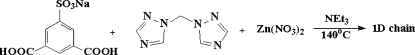
Reaction scheme

## supplementary crystallographic information

### Comment

Organic-inorganic hybrid materials have obtained extensive attention due to not only the structural diversity but also their attractive properties, such as catalytic activity, magnetism, photochemical activity and electrical chemistry (Ishikava *et al.*, 2003). One of the key steps for preparation of polymeric transition metal complexes is to select the multidentate bridging ligands or mixed multidentate ligands (Biradha *et al.*, 2006). 5-Sulfoisophthalic acid as a kind of multi-carboxylic ligand is a good bridging ligand, but it has been less explored for the synthesis. On the other hand, 1,4-bis(1,2,4-triazol-1-yl)methane (abbreviated as btrm) is a flexible ligand. In this contribution, we describe the Zn(II) metal-organic frameworks constructed from the rigid multi-carboxylic ligand sip and flexible btrm ligand. A new complex {[Zn(btrp)(sip)][Zn_0.5_(H_2_O)]}_n_ was fabricated.

The title compound possesses a dinuclear structure with the asymmetric unit containing one crystallographically unique Zn^2+^ ion, one btrm ligand, one sip ligand and half of one free Zn(H_2_O)_6_^2+^ ion. As viewed in Fig. 1, Zn1 is five-coordinated in a highly distorted trigonal biyramid coordination sphere that is defined by two different nitrogen atoms from two btrm ligands and three carboxylic oxygen atoms. Both btrm and sip adopt two connected mode. Every sip ligand links two Zn(II) atoms to construct a one-dimensional chain, two such chains are bridged by *cis*-btrm ligands to produce a one-dimensional tubelike chain (Fig. 2).  Noteworthily, the one-dimensional chains carry negative charges, whereas the free Zn(H_2_O)_6_^2+^ ion show positive electricity. Through weak classical hydrogen-bonding interactions as well as the electrostatic interactions, the Zn(H_2_O)_6_^2+^ ions are connected with the one-dimensional tubelike chain.

### Experimental

A mixture of [Zn(NO_3_)_2_]6H_2_O (148 mg, 0.5 mmol), NaH_2_sip (135 mg, 0.5 mmol), btrm (68 mg, 0.5 mmol), triethylamine (1.0 mmol), H_2_O (12 ml) was added into a Parr Teflon-lined stainless steel vessel, and then the vessel was sealed and heated to 413 K, kept for 3 days. After that the autoclave was cooled to room temperature at a rate of 1.5 °K/h. The title compound was filtered off, washed with distilled water and dried in air (yield 65% based on Zn). Analysis, calculated for C_13_H_18_N_6_O_11.5_SZn_1.5_: *C* 27.29, H 3.17, N 14.68; found: C 27.01, H 3.58, N 14.88%.

### Refinement

After the non-hydrogen atoms of the cation and anion had been located, a number of peaks remained in the difference electron density. We have assigned these as water of solvation, O11 and O12. As a result of the large Ueq on O12 this atom was assigned an occupation number of 0.5 which is consistent with the C, H and N elemental analyses. It was not possible to locate the hydrogen atoms around O12, but those around O11 were located from difference Fourier maps and further refined by using geometrical restraints. Several small, but significant, peaks of around 1.5 e/A^3^ remain in the neighborhood of the the cation.

H atoms were positioned geometrically with O—H = 0.86 Å, C—H = 0.93 and 0.97 Å for aromatic and methylene H atoms, respectivly, and constrained to ride on their parent atoms, and included in the final cycles of refinement using a riding model, with *U*_iso_(H) = 1.2Ueq(C).

### Crystal data

**Table:**

[Zn(H_2_O)_6_]_0.5_[Zn(C_8_H_3_O_7_S)(C_5_H_6_N_6_)]·1.5H_2_O	*F*(000) = 1160
*M**_r_* = 571.48	*D*_x_ = 1.900 Mg m^−^^3^
Monoclinic, *P*2_1_/*c*	Mo *K*α radiation, λ = 0.71073 Å
Hall symbol: -P 2ybc	Cell parameters from 4538 reflections
*a* = 10.2611 (3) Å	θ = 2.4–25.0°
*b* = 16.9967 (4) Å	µ = 2.00 mm^−^^1^
*c* = 11.4808 (3) Å	*T* = 293 K
β = 93.812 (2)°	BLOCK, colourless
*V* = 1997.88 (9) Å^3^	0.23 × 0.15 × 0.14 mm
*Z* = 4	

### Data collection

**Table:**

Bruker SuperNova Eos diffractometer	3524 independent reflections
Radiation source: SuperNova (Mo) X-ray Source	2763 reflections with *I* > 2σ(*I*)
mirror	*R*_int_ = 0.030
Detector resolution: 16.2116 pixels mm^-1^	θ_max_ = 25.0°, θ_min_ = 2.4°
ω scans	*h* = −12→10
Absorption correction: multi-scan (*SADABS*; Bruker, 1997)	*k* = −20→19
*T*_min_ = 0.657, *T*_max_ = 0.767	*l* = −8→13
7740 measured reflections	

### Refinement

**Table:**

Refinement on *F*^2^	Primary atom site location: structure-invariant direct methods
Least-squares matrix: full	Secondary atom site location: difference Fourier map
*R*[*F*^2^ > 2σ(*F*^2^)] = 0.049	Hydrogen site location: inferred from neighbouring sites
*wR*(*F*^2^) = 0.155	H-atom parameters constrained
*S* = 1.11	*w* = 1/[σ^2^(*F*_o_^2^) + (0.0939*P*)^2^ + 1.3769*P*] where *P* = (*F*_o_^2^ + 2*F*_c_^2^)/3
3524 reflections	(Δ/σ)_max_ = 0.001
304 parameters	Δρ_max_ = 1.67 e Å^−^^3^
30 restraints	Δρ_min_ = −0.90 e Å^−^^3^

### Special details

**Table:**

Geometry. All esds (except the esd in the dihedral angle between two l.s. planes) are estimated using the full covariance matrix. The cell esds are taken into account individually in the estimation of esds in distances, angles and torsion angles; correlations between esds in cell parameters are only used when they are defined by crystal symmetry. An approximate (isotropic) treatment of cell esds is used for estimating esds involving l.s. planes.
Refinement. Refinement of *F*^2^ against ALL reflections. The weighted *R*-factor *wR* and goodness of fit *S* are based on *F*^2^, conventional *R*-factors *R* are based on *F*, with *F* set to zero for negative *F*^2^. The threshold expression of *F*^2^ > σ(*F*^2^) is used only for calculating *R*-factors(gt) *etc*. and is not relevant to the choice of reflections for refinement. *R*-factors based on *F*^2^ are statistically about twice as large as those based on *F*, and *R*- factors based on ALL data will be even larger.

### Fractional atomic coordinates and isotropic or equivalent isotropic displacement parameters (Å^2^)

**Table:**

	*x*	*y*	*z*	*U*_iso_*/*U*_eq_	Occ. (<1)
Zn1	−0.05241 (5)	0.44539 (3)	0.65524 (5)	0.0173 (2)	
Zn2	0.5000	0.5000	1.0000	0.0242 (3)	
C1	−0.1029 (5)	0.5844 (3)	0.8059 (5)	0.0223 (12)	
H1	−0.1689	0.5583	0.8420	0.027*	
C2	0.0432 (6)	0.6157 (3)	0.6959 (5)	0.0290 (13)	
H2	0.1013	0.6129	0.6372	0.035*	
C3	−0.0915 (6)	0.7063 (3)	0.9310 (4)	0.0222 (12)	
H3A	−0.0726	0.7606	0.9123	0.027*	
H3B	−0.1841	0.7020	0.9418	0.027*	
C4	−0.0526 (5)	0.6384 (3)	1.1245 (5)	0.0226 (12)	
H4	−0.1355	0.6172	1.1301	0.027*	
C5	0.1439 (5)	0.6712 (3)	1.1562 (5)	0.0263 (13)	
H5	0.2270	0.6761	1.1930	0.032*	
C6	0.3270 (5)	0.4047 (3)	0.5589 (4)	0.0164 (11)	
C7	0.3175 (5)	0.3543 (3)	0.4624 (4)	0.0177 (11)	
H7	0.2362	0.3380	0.4308	0.021*	
C8	0.4303 (5)	0.3289 (3)	0.4144 (4)	0.0166 (11)	
C9	0.5517 (5)	0.3537 (3)	0.4584 (4)	0.0178 (11)	
H9	0.6265	0.3364	0.4247	0.021*	
C10	0.5614 (5)	0.4045 (3)	0.5531 (4)	0.0156 (11)	
C11	0.4512 (5)	0.4286 (3)	0.6035 (4)	0.0168 (11)	
H11	0.4587	0.4614	0.6685	0.020*	
C12	0.2081 (5)	0.4314 (3)	0.6171 (5)	0.0191 (11)	
C13	0.6940 (5)	0.4296 (3)	0.6051 (4)	0.0173 (11)	
N1	−0.0406 (4)	0.5563 (2)	0.7185 (4)	0.0198 (10)	
N2	−0.0580 (4)	0.6556 (2)	0.8351 (4)	0.0201 (10)	
N3	0.0351 (5)	0.6768 (3)	0.7636 (4)	0.0308 (12)	
N4	−0.0173 (4)	0.6839 (2)	1.0379 (4)	0.0199 (9)	
N5	0.1097 (5)	0.7049 (3)	1.0573 (4)	0.0285 (11)	
N6	0.0471 (4)	0.6278 (2)	1.2015 (4)	0.0210 (10)	
O1	0.0957 (3)	0.4135 (2)	0.5658 (3)	0.0265 (9)	
O2	0.2190 (3)	0.4662 (2)	0.7126 (3)	0.0258 (9)	
O3	0.7036 (3)	0.4664 (2)	0.6994 (3)	0.0263 (9)	
O4	0.7944 (3)	0.4118 (2)	0.5511 (3)	0.0245 (9)	
O5	0.3252 (4)	0.1997 (2)	0.3349 (4)	0.0410 (11)	
O6	0.3664 (4)	0.3024 (2)	0.1958 (4)	0.0401 (11)	
O7	0.5457 (4)	0.2277 (2)	0.2870 (4)	0.0391 (11)	
O8	0.3948 (5)	0.5718 (3)	1.1028 (5)	0.0574 (14)	
H8A	0.3892	0.5761	1.1773	0.028*	
H8B	0.3861	0.6192	1.0761	0.028*	
O9	0.6329 (5)	0.5931 (3)	0.9813 (5)	0.0526 (13)	
H9A	0.6046	0.6261	0.9278	0.028*	
H9B	0.6511	0.6216	1.0425	0.028*	
O10	0.3976 (5)	0.5446 (3)	0.8539 (5)	0.0565 (14)	
H10A	0.3721	0.5071	0.8081	0.028*	
H10B	0.3284	0.5688	0.8745	0.028*	
O11	0.3350 (5)	0.6924 (3)	0.9006 (4)	0.0494 (12)	
H11A	0.3859	0.7322	0.8968	0.028*	
H11B	0.2577	0.7116	0.9057	0.028*	
O12	0.133 (2)	0.5302 (11)	0.9610 (18)	0.138 (6)	0.50
S1	0.41635 (13)	0.25941 (8)	0.29848 (12)	0.0236 (3)	

### Atomic displacement parameters (Å^2^)

**Table:**

	*U*^11^	*U*^22^	*U*^33^	*U*^12^	*U*^13^	*U*^23^
Zn1	0.0120 (3)	0.0235 (3)	0.0165 (4)	−0.0001 (2)	0.0004 (2)	−0.0058 (2)
Zn2	0.0253 (5)	0.0277 (5)	0.0200 (5)	−0.0025 (4)	0.0037 (4)	−0.0067 (4)
C1	0.021 (3)	0.020 (3)	0.026 (3)	−0.003 (2)	0.005 (2)	−0.003 (2)
C2	0.034 (3)	0.031 (3)	0.024 (3)	−0.010 (3)	0.012 (3)	0.000 (3)
C3	0.031 (3)	0.020 (3)	0.016 (3)	0.003 (2)	0.002 (2)	−0.004 (2)
C4	0.020 (3)	0.025 (3)	0.023 (3)	0.000 (2)	0.000 (2)	−0.003 (2)
C5	0.019 (3)	0.031 (3)	0.029 (3)	−0.005 (2)	0.002 (2)	−0.002 (3)
C6	0.013 (3)	0.018 (2)	0.018 (3)	0.002 (2)	0.002 (2)	0.004 (2)
C7	0.010 (3)	0.022 (3)	0.020 (3)	−0.004 (2)	−0.003 (2)	0.002 (2)
C8	0.018 (3)	0.016 (2)	0.016 (3)	−0.002 (2)	0.000 (2)	−0.001 (2)
C9	0.015 (3)	0.019 (2)	0.020 (3)	−0.002 (2)	0.004 (2)	−0.002 (2)
C10	0.012 (3)	0.021 (2)	0.013 (3)	0.000 (2)	−0.001 (2)	0.004 (2)
C11	0.016 (3)	0.019 (2)	0.014 (3)	0.000 (2)	−0.002 (2)	−0.002 (2)
C12	0.013 (3)	0.023 (3)	0.022 (3)	0.000 (2)	0.003 (2)	0.004 (2)
C13	0.015 (3)	0.022 (2)	0.015 (3)	−0.002 (2)	0.002 (2)	0.007 (2)
N1	0.018 (2)	0.023 (2)	0.019 (2)	0.0011 (18)	0.0012 (18)	−0.0030 (18)
N2	0.023 (2)	0.020 (2)	0.018 (2)	0.0012 (18)	0.0054 (19)	−0.0008 (18)
N3	0.044 (3)	0.027 (2)	0.023 (3)	−0.014 (2)	0.012 (2)	−0.004 (2)
N4	0.018 (2)	0.022 (2)	0.020 (2)	0.0000 (18)	0.0022 (18)	−0.0052 (19)
N5	0.025 (3)	0.030 (2)	0.031 (3)	−0.004 (2)	0.004 (2)	−0.001 (2)
N6	0.019 (2)	0.024 (2)	0.020 (2)	0.0025 (19)	0.0004 (18)	−0.0021 (19)
O1	0.0118 (19)	0.042 (2)	0.025 (2)	0.0029 (16)	0.0014 (16)	−0.0110 (18)
O2	0.017 (2)	0.037 (2)	0.024 (2)	0.0014 (17)	0.0025 (16)	−0.0123 (18)
O3	0.016 (2)	0.043 (2)	0.020 (2)	−0.0070 (17)	0.0008 (15)	−0.0093 (18)
O4	0.0080 (18)	0.041 (2)	0.025 (2)	0.0024 (16)	0.0027 (15)	−0.0077 (17)
O5	0.041 (3)	0.032 (2)	0.051 (3)	−0.019 (2)	0.009 (2)	−0.009 (2)
O6	0.044 (3)	0.052 (3)	0.023 (2)	−0.001 (2)	−0.0047 (19)	−0.008 (2)
O7	0.026 (2)	0.050 (2)	0.042 (3)	0.0041 (19)	0.0052 (19)	−0.022 (2)
O8	0.063 (2)	0.0562 (19)	0.054 (2)	0.0053 (17)	0.0110 (17)	−0.0018 (16)
O9	0.052 (2)	0.0526 (19)	0.053 (2)	−0.0027 (16)	−0.0012 (16)	−0.0009 (16)
O10	0.059 (2)	0.0541 (19)	0.055 (2)	0.0054 (16)	−0.0057 (17)	−0.0066 (16)
O11	0.0467 (19)	0.0461 (17)	0.057 (2)	−0.0014 (16)	0.0128 (16)	−0.0003 (16)
O12	0.138 (6)	0.138 (6)	0.138 (6)	0.000 (2)	0.009 (2)	0.000 (2)
S1	0.0189 (7)	0.0269 (7)	0.0250 (8)	−0.0024 (6)	0.0008 (6)	−0.0094 (6)

### Geometric parameters (Å, °)

**Table:**

Zn1—O1	1.967 (4)	C6—C11	1.402 (7)
Zn1—O4^i^	1.994 (4)	C6—C12	1.500 (7)
Zn1—N1	2.020 (4)	C7—C8	1.384 (7)
Zn1—N6^ii^	2.059 (4)	C7—H7	0.9300
Zn1—O3^i^	2.611 (4)	C8—C9	1.378 (7)
Zn2—O8^iii^	2.053 (5)	C8—S1	1.778 (5)
Zn2—O8	2.053 (5)	C9—C10	1.388 (7)
Zn2—O10^iii^	2.063 (5)	C9—H9	0.9300
Zn2—O10	2.063 (5)	C10—C11	1.367 (7)
Zn2—O9	2.109 (5)	C10—C13	1.510 (7)
Zn2—O9^iii^	2.109 (5)	C11—H11	0.9300
C1—N1	1.315 (7)	C12—O2	1.244 (6)
C1—N2	1.331 (6)	C12—O1	1.296 (6)
C1—H1	0.9300	C13—O3	1.248 (6)
C2—N3	1.304 (7)	C13—O4	1.274 (6)
C2—N1	1.363 (7)	N2—N3	1.348 (6)
C2—H2	0.9300	N4—N5	1.355 (6)
C3—N4	1.451 (7)	O5—S1	1.459 (4)
C3—N2	1.457 (6)	O6—S1	1.452 (4)
C3—H3A	0.9700	O7—S1	1.446 (4)
C3—H3B	0.9700	O8—H8A	0.8647
C4—N6	1.319 (7)	O8—H8B	0.8647
C4—N4	1.329 (7)	O9—H9A	0.8672
C4—H4	0.9300	O9—H9B	0.8636
C5—N5	1.299 (7)	O10—H10A	0.8566
C5—N6	1.367 (7)	O10—H10B	0.8672
C5—H5	0.9300	O11—H11A	0.8576
C6—C7	1.398 (7)	O11—H11B	0.8636
			
O1—Zn1—O4^i^	102.35 (15)	C8—C9—H9	120.2
O1—Zn1—N1	114.53 (17)	C10—C9—H9	120.2
O4^i^—Zn1—N1	120.59 (16)	C11—C10—C9	120.0 (5)
O1—Zn1—N6^ii^	105.67 (16)	C11—C10—C13	119.8 (4)
O4^i^—Zn1—N6^ii^	106.46 (16)	C9—C10—C13	120.1 (4)
N1—Zn1—N6^ii^	106.17 (17)	C10—C11—C6	121.1 (5)
O1—Zn1—O3^i^	157.39 (13)	C10—C11—H11	119.5
O4^i^—Zn1—O3^i^	55.08 (13)	C6—C11—H11	119.5
N1—Zn1—O3^i^	80.70 (15)	O2—C12—O1	122.5 (5)
N6^ii^—Zn1—O3^i^	84.39 (15)	O2—C12—C6	120.6 (5)
O8^iii^—Zn2—O10^iii^	89.4 (2)	O1—C12—C6	116.9 (5)
O8—Zn2—O10^iii^	90.6 (2)	O3—C13—O4	121.4 (5)
O8^iii^—Zn2—O9	91.1 (2)	O3—C13—C10	120.1 (5)
O8—Zn2—O9	88.9 (2)	O4—C13—C10	118.5 (4)
O10^iii^—Zn2—O9	93.5 (2)	C1—N1—C2	102.7 (4)
O10—Zn2—O9	86.5 (2)	C1—N1—Zn1	126.3 (4)
O8^iii^—Zn2—O9^iii^	88.9 (2)	C2—N1—Zn1	130.3 (4)
O8—Zn2—O9^iii^	91.1 (2)	C1—N2—N3	109.7 (4)
O10^iii^—Zn2—O9^iii^	86.5 (2)	C1—N2—C3	129.3 (5)
O10—Zn2—O9^iii^	93.5 (2)	N3—N2—C3	120.9 (4)
N1—C1—N2	110.2 (5)	C2—N3—N2	103.0 (4)
N1—C1—H1	124.9	C4—N4—N5	109.7 (4)
N2—C1—H1	124.9	C4—N4—C3	129.2 (5)
N3—C2—N1	114.3 (5)	N5—N4—C3	121.0 (4)
N3—C2—H2	122.9	C5—N5—N4	103.2 (4)
N1—C2—H2	122.9	C4—N6—C5	102.8 (5)
N4—C3—N2	110.3 (4)	C4—N6—Zn1^ii^	126.6 (4)
N4—C3—H3A	109.6	C5—N6—Zn1^ii^	130.4 (4)
N2—C3—H3A	109.6	C12—O1—Zn1	113.1 (3)
N4—C3—H3B	109.6	C13—O3—Zn1^iv^	77.6 (3)
N2—C3—H3B	109.6	C13—O4—Zn1^iv^	105.7 (3)
H3A—C3—H3B	108.1	Zn2—O8—H8A	133.7
N6—C4—N4	110.1 (5)	Zn2—O8—H8B	113.3
N6—C4—H4	125.0	H8A—O8—H8B	105.1
N4—C4—H4	125.0	Zn2—O9—H9A	111.6
N5—C5—N6	114.2 (5)	Zn2—O9—H9B	116.4
N5—C5—H5	122.9	H9A—O9—H9B	104.9
N6—C5—H5	122.9	Zn2—O10—H10A	110.1
C7—C6—C11	118.7 (5)	Zn2—O10—H10B	109.5
C7—C6—C12	121.5 (4)	H10A—O10—H10B	107.3
C11—C6—C12	119.7 (4)	H11A—O11—H11B	105.8
C8—C7—C6	119.4 (4)	O7—S1—O6	113.0 (3)
C8—C7—H7	120.3	O7—S1—O5	112.1 (3)
C6—C7—H7	120.3	O6—S1—O5	112.6 (3)
C9—C8—C7	121.2 (5)	O7—S1—C8	106.7 (2)
C9—C8—S1	120.2 (4)	O6—S1—C8	106.3 (2)
C7—C8—S1	118.6 (4)	O5—S1—C8	105.6 (2)
C8—C9—C10	119.6 (5)		

Symmetry codes: (i) *x*−1, *y*, *z*; (ii) −*x*, −*y*+1, −*z*+2; (iii) −*x*+1, −*y*+1, −*z*+2; (iv) *x*+1, *y*, *z*.

### Hydrogen-bond geometry (Å, °)

**Table:**

*D*—H···*A*	*D*—H	H···*A*	*D*···*A*	*D*—H···*A*
O8—H8A···O3^iii^	0.86	1.90	2.628 (7)	141
O8—H8B···O11	0.86	2.40	3.126 (7)	143
O8—H8B···O7^v^	0.86	2.49	2.983 (7)	117
O9—H9A···O6^vi^	0.86	1.91	2.700 (7)	151
O9—H9B···O5^v^	0.86	1.94	2.794 (7)	170
O10—H10A···O2	0.86	1.98	2.716 (7)	143
O10—H10B···O11	0.86	2.12	2.656 (7)	119
O10—H10B···O12	0.86	2.39	3.068 (7)	135
O11—H11A···O7^vi^	0.86	2.37	2.887 (7)	119
O11—H11B···N5	0.86	2.39	3.029 (7)	131

Symmetry codes: (iii) −*x*+1, −*y*+1, −*z*+2; (v) −*x*+1, *y*+1/2, −*z*+3/2; (vi) −*x*+1, −*y*+1, −*z*+1.
